# Notes

**Published:** 1896-02

**Authors:** 


### Notes.

Medical Climatology.—A national meeting of the "World's Congress of Medico-Climatology," will be held in San Antonio, Texas, beginning February 20, 1896, and continuing for three days. All physicians in good standing are invited to attend. The address of the corresponding secretary, to whom all inquiries should be addressed, is W. S. Rowley, M. D., Menger Hotel, San Antonio, Texas.

---

Dr. Charles S. Webb has resigned the chair of practice of medicine in the Southern Medical College, and Dr. James B. Baird has been elected. The vacancy on the State Board of Medical Examiners, occasioned by the necessity of Dr. Baird's resignation to accept the chair at the college, has been filled by the appointment of Dr. John C. Olmsted, of Atlanta.

Other changes at the Southern Medical College: Judge Howard Van Epps has been elected president of the Board of Trustees; Dr. J. McF. Gaston, vice president; Col. W. W. McAfee, treasurer; and Dr. Wm. Perrin Nicolson, president of the faculty.

---

At the meeting of the Atlanta Medical Society, held Tuesday, January 21, the following committee was appointed to arrange for the approaching meeting of the American Medical Association: Dr. W. F. Westmoreland, chairman; Dr. J. McF Gaston, secretary and treasurer; Drs. Hunter P. Cooper, W. S. Elkin, R. R. Kime, L. B. Grandy, W. C. Jarnagan, Louis H. Jones, George H. Noble, G. G. Roy, R. B. Ridley, J. M. Crawford, L. Austen, W. P. Nicolson, J. A. Fielder, A. W. Calhoun, J C. Olmsted, L. P. Stephens, W. S. Kendrick, W. L. Champion, J. S. Todd, Bernard Wolff, Hugh Hagan, C. D. Hurt, and V. O. Hardon.

